# Das RCEP-Abkommen und dessen Bedeutung für die EU

**DOI:** 10.1007/s10273-021-2938-x

**Published:** 2021-06-22

**Authors:** Michael Frenkel, Tuyet Ngo

**Affiliations:** Makroökonomik und Internationale Wirtschaftsbeziehungen, WHU - Otto Beisheim School of Management, Erkrather Str. 224a, 40244 Düssedorf, Deutschland

**Keywords:** F02, F15, F53

## Abstract

Im November 2020 wurde das RCEP-Abkommen als größtes Freihandelsabkommen der Welt von Brunei, Kambodscha, Indonesien, Laos, Malaysia, Myanmar, den Philippinen, Singapur, Thailand, Vietnam sowie Australien, China, Japan, Neuseeland und Südkorea unterzeichnet. Bis Ende 2022 soll das Abkommen ratifiziert sein und in Kraft treten. Zusammen entfallen auf die Mitgliedsländer 25 % des internationalen Waren- und Dienstleistungsverkehrs. Es stellt sich die Frage, welche positiven und negativen Auswirkungen das Freihandelsabkommen für die Mitgliedsländer sowie die EU haben wird.

## Abstract (en)

The Regional Comprehensive Economic Partnership (RCEP) is a multilateral free trade agreement (FTA) between the member countries of the Association of Southeast Asian Nations (ASEAN) and five Asia-Pacific countries, with which ASEAN has existing FTAs in the Asia-Pacific region. It was initiated with the aim to broaden and deepen regional trade and investment among the signatories. The agreement was finalised and signed in November 2020. It is now the largest free trade agreement in the world. The article examines the expected effects and their implications for the EU. It also outlines strategic ways in which the EU could respond to this new agreement.

Das Regional Comprehensive Economic Partnership (RCEP)-Abkommen ist ein multilaterales Freihandelsabkommen zwischen den Mitgliedsländern des Verbands Südostasiatischer Nationen (ASEAN) und fünf asiatisch-pazifischen Ländern, mit denen ASEAN bestehende Freihandelsabkommen im asiatisch-pazifischen Raum unterhält. Es wurde im November 2011 von den ASEAN-Ländern vorgeschlagen mit dem Ziel, den regionalen Handel und die Investitionen zwischen den Unterzeichnenden zu erweitern und zu vertiefen. Die Verhandlungen zu dem Abkommen begannen offiziell im November 2012. Nach acht Jahren Verhandlungen mit 31 Verhandlungsrunden und 18 Ministertreffen wurde das Abkommen im November 2020 während des 37. ASEAN-Gipfels fertiggestellt und unterzeichnet. Das RCEP-Abkommen ist nun das größte Freihandelsabkommen der Welt, deutlich vor der Europäischen Union (EU), dem Nordamerikanischen Freihandelsabkommen (NAFTA, jetzt USMCA) und dem Comprehensive and Progressive Agreement for Trans-Pacific Partnership (CPTPP). Die RCEP-Unterzeichner sind die zehn ASEAN-Mitglieder (Brunei, Kambodscha, Indonesien, Laos, Malaysia, Myanmar, die Philippinen, Singapur, Thailand, Vietnam) sowie Australien, China, Japan, Neuseeland und Südkorea. Indien war ursprünglich Verhandlungsteilnehmer, entschied dann aber gegen Ende der Verhandlungsphase, sich zurückzuziehen, da es den Wettbewerb vor allem durch chinesische Waren fürchtete. Insofern werden die Mitgliedsländer des RCEP-Blocks oft als ASEAN Plus 6 ohne Indien bezeichnet. Es wird erwartet, dass der Prozess der Ratifizierung und des Inkrafttretens bis Ende 2022 abgeschlossen ist.

**Tuyet Ngo** ist wissenschaftliche Mitarbeiterin an der WHU – Otto Beisheim School of Management in Vallendar/Koblenz.

## Warum ist das Freihandelsabkommen RCEP wichtig?

Auf die RCEP-Mitgliedsländer entfallen etwa 25 % des internationalen Waren- und Dienstleistungsverkehrs und fast ein Drittel des weltweiten BIP, wobei diese Zahl bis 2030 voraussichtlich 50 % erreichen wird. Der RCEP-Block deckt einen Markt von fast 2,3 Mrd. Menschen ab, was 30 % der Weltbevölkerung entspricht, und dient als gigantische Handelsplattform für Unternehmen, nicht nur innerhalb der Region, sondern auch von außerhalb.[1]

Seit einiger Zeit ist erkennbar, dass sich das globale Wirtschaftszentrum mehr und mehr nach Asien verlagert, wobei China die wichtigste Rolle spielt. Gerade in der Zeit, in der die COVID-19-Pandemie noch vorherrscht, entsteht das RCEP-Abkommen als eine wesentliche Antwort auf die Notwendigkeit der Umstrukturierung globaler Liefer-

1 Die Daten zum RCEP-Block sind aus New Zealand Ministry of Foreign Affairs & Trade, FTA Implementation Unit (2020); Projektionen sind aus Neumann und Rajanayagam (2020).

Tabelle 1
**Zusammenfassung der wichtigsten Ergebnisse des RCEP-Abkommens**

| | |
|---|---|
| Warenhandel | Hierin liegt der ehrgeizigste Teil des RCEP-Abkommens. Innerhalb von 20 Jahren sollen mindestens 90 % der Zölle auf Waren, die in der Region gehandelt werden, beseitigt werden. Die vereinbarten Regelungen gewähren den Mitgliedsländern neue und wichtige Marktzugänge in Bezug auf Waren, vor allem denjenigen, die keine Freihandelsabkommen mit anderen Ländern des RCEP-Blocks haben (z. B. zwischen China und Japan, Japan und Südkorea sowie Japan und Neuseeland). Zur Förderung der Handelsliberalisierung werden auch einige nichttarifäre Maßnahmen festgelegt, wie z. B. die Verbesserung der Transparenz bei der Verwaltung von Importlizenzverfahren und der Anwendung von Gebühren und Formalitäten im Zusammenhang mit der Ein- und Ausfuhr. |
| Ursprungsregeln | Durch produktspezifische Regeln (Product-Specific Rules) für die Bestimmung der Ursprungslandeigenschaft von Waren einschließlich Regeln für die Verpackung für den Transport und die Behandlung von Teilen und Zubehör soll sichergestellt werden, dass Waren, die innerhalb der Region gehandelt werden, ihre Ursprungseigenschaft nicht verlieren. Hiermit können Wertschöpfungsketten über Ländergrenzen innerhalb des RCEP-Blocks organisiert werden. |
| Zollverfahren und Handelserleichterung | Dieses Regelwerk vereinfacht die Zollverfahren und sieht weitere handelsfördernde Maßnahmen vor, wie die Freigabe von Waren aus der Zoll- und Grenzkontrolle innerhalb von sechs Stunden und die Zollabfertigung innerhalb von 48 Stunden nach Ankunft der Waren. |
| Dienstleistungshandel | Diese Bestimmungen zielen darauf ab, den Handel mit Dienstleistungen innerhalb der Region zu verbessern, indem sie Regeln zum Marktzugang, zur Inländerbehandlung sowie zur Meistbegünstigung festlegen. Sie konzentrieren sich jedoch hauptsächlich auf den Handel mit Finanz-, Telekommunikations- und professionellen Dienstleistungen. Andere Arten von Dienstleistungen werden nicht ausdrücklich erwähnt oder sind derzeit noch Gegenstand von Verhandlungen. |
| Investitionen | Die Bestimmungen in diesem Bereich umfassen Kernregeln zum Investitionsschutz wie Entschädigung für Enteignung, konfliktbedingte Verluste und den freien Transfer von Investitionskapital. Die Regeln zur Investor-Staat-Streitbeilegung (ISDS) sind jedoch noch nicht abgeschlossen und verbleiben unter einem „eingebauten Arbeitsprogramm" für zukünftige Verhandlungen. |
| Geistige Eigentumsrechte | Neben den Regeln zum Standardschutz geistiger Eigentumsrechte erstrecken sich diese Bestimmungen auch auf das digitale Umfeld, einschließlich Klangmarken und gewerblicher Muster. Alle Mitgliedsländer sind außerdem verpflichtet, sich an eine Regelung zum Schutz der geografischen Indikatoren zu halten. |
| E-Commerce | Die Bestimmungen in diesem Bereich ermutigen die Mitgliedsländer, elektronische Mittel für Handelsverfahren einzuführen und diese parallel zum traditionellen Rechtsrahmen zu nutzen. Sie bieten weiteren Schutz für personenbezogene Daten von Anbietern und Verbrauchern, die den elektronischen Handel nutzen, und behandelt verschiedene Fragen im Zusammenhang mit dem grenzüberschreitenden Datentransfer. |

Quelle: eigene Zusammenstellung und Kommentare basierend auf RCEP (2020), ASEAN (2020) und Australian Department of Foreign Affairs and Trade (2020a).

ketten durch die Förderung der Regionalisierung, insbesondere im Fertigungssektor.

## Der Umfang des RCEP-Abkommens

Das oberste Ziel des RCEP-Abkommens ist die Beseitigung von Zöllen und Kontingenten, um damit den regionalen Handel zu fördern. Etwa 65 % der Waren können in der Region ohne Zölle gehandelt werden, wenn das Abkommen in Kraft tritt und die meisten der verbleibenden Zölle innerhalb der nächsten zwei Jahrzehnte schrittweise abgeschafft werden sollen. Insgesamt besteht das RCEP-Abkommen aus 20 Bestimmungen, die wichtige Aspekte der multilateralen Beziehungen wie Warenhandel, Ursprungsregeln, Zollverfahren und Handelserleichterungen, Handel mit Dienstleistungen, Investitionen, elektronischer Handel und geistiges Eigentum abdecken (vgl. Tabelle 1).

Das Abkommen umfasst auch Bestimmungen zur Streitschlichtung bei Auslegung und Anwendung der Vertragsbestimmungen und zu handelspolitischen Antidumping- und anderen Schutzmaßnahmen. Im Grundsatz sind die Mechanismen an die der WTO angelehnt. Die festgelegten Prozesse implizieren, dass in jeder Phase Anreize für einvernehmliche Lösungen bestehen (BDI, 2020).

Im Vergleich zu bestehenden Freihandelsabkommen der EU mit ihren Handelspartnern ist das RCEP-Abkommen weniger umfassend. Obwohl beispielsweise der Handel mit Dienstleistungen eine der wichtigsten Bestimmungen darstellt, ist der Umfang für diesen Sektor nicht sehr ambitioniert. Insbesondere zielt das Regelwerk für den Handel mit Dienstleistungen nur darauf ab, ausländischen Dienstleistern einen besseren Marktzugang mit weniger Unsicherheiten zu gewähren. Es ist jedoch auf drei Arten von Dienstleistungen beschränkt, nämlich Finanzdienstleistungen, Telekommunikation und freiberufliche Dienstleistungen, sodass andere Dienstleistungen weniger Liberalisierung erfahren. Im Hinblick auf den digitalen Handel fördert das RCEP-Abkommen weder regionale Standards, noch schränkt es die Möglichkeiten von Regierungen ein, die Offenlegung von Quellcodes von IT-Unternehmen zu verlangen. Darüber hinaus deckt es einige wichtige Merkmale dessen, was oft als „besserer“ Handel bezeichnet wird, nicht ab, wie etwa Sozialstandards, Umweltschutz und Arbeitsrechte. Die Tatsache, dass das RCEP-Abkommen keinen Konsens zu nachhaltiger Entwicklung und zur Einschränkung der Rechte von Regierungen im Zusammenhang mit den Quellcodes von IT-Unternehmen erzielen konnte, ist vermutlich auf das unterschiedliche institutionelle und wirtschaftliche Entwicklungsniveau der Mitgliedsländer zurückzuführen. Während einige der 15 RCEP-Unterzeichnerländer zu den Ländern mit hohen Einkommen gehören (z.B. Australien, Japan, Neuseeland, Singapur und Südkorea), gehören einige Länder zur Gruppe mit dem niedrigsten Einkommen (z.B. Kambodscha, Laos und Myanmar). Darüber hinaus haben einige Länder eher dezentralisierte Wirtschaftsstrukturen, während andere eher zentralisierte Wirtschaftsstrukturen haben. Daher würden umfassendere Regeln mehr Zeit und Anstrengungen für weitere Verhandlungen der RCEP-Mitgliedsländer erfordern.

## Erwartete Auswirkungen des RCEP-Abkommens auf einzelne Mitgliedsländer

Auch wenn das RCEP-Abkommen von den ASEAN-Staaten initiiert und angeführt wurde, scheint China aus mehreren Gründen der größte Gewinner dieses Abkommens zu sein. *Erstens* können chinesische Waren nun mit Vorzugszöllen auf den Markt Japans, der drittgrößten Volkswirtschaft der Welt, gelangen, was für China in der Vergangenheit aufgrund seiner politischen und historischen Konflikte mit Japan nicht möglich war. *Zweitens* hat China durch die regionale Handelsliberalisierung, die durch das RCEP-Abkommen erreicht wird, mehr Möglichkeiten, seinen wirtschaftlichen Einfluss in der asiatisch-pazifischen Region zu vergrößern, insbesondere da sich die USA von Handelsabkommen mit Ländergruppen in dieser Region zurückgezogen haben.[2] *Drittens* ist zu erwarten, dass die Einbindung der größten Volkswirtschaft Asiens die wirtschaftliche Integration und Verflechtung der gesamten Region beschleunigen wird. *Viertens* kann die Tatsache, dass westliche Länder wie Australien, Japan, Südkorea und Neuseeland stärkere Verbindungen zu China aufbauen, als ein großer Erfolg Chinas angesehen werden, weil diese Länder wirtschaftlich näher an China heranrücken und damit weg von den USA. Implizit signalisieren diese Länder mit dem RCEP-Abkommen auch ihre Skepsis gegenüber der von den USA propagierten Strategie der technologischen und wirtschaftlichen Abkopplung von China (Helmold und Dathe, 2020).

Obwohl die ASEAN-Mitgliedsländer über die militärische Präsenz Chinas im Südchinesischen Meer besorgt sind, waren sie bereit, das RCEP-Abkommen zu unterzeichnen, weil sie sich davon eine Beschleunigung ihrer wirtschaftlichen Entwicklung versprechen. Das RCEP-Abkommen, das über die bestehenden sogenannten ASEAN-Plus-Eins-Freihandelsabkommen hinausgeht – diese bestehen zwischen AEA-China (ACFTA), ASEAN-Korea (AKFTA) und ASEAN-Japan (AJFTA) –, wird den Unternehmen in den RCEP-Ländern einen leichteren Zugang zu einigen der größten Märkte der Welt ermöglichen. Die Unternehmen können z.B. von den standardisierten Ursprungsregeln profitieren, da diese es den Firmen ermöglichen, ihre Produkte über die Länder der RCEP-Region hinweg zu versenden, ohne dass sie in jeder Produktionsstufe und in jedem Land, das sie durchqueren, Schwierigkeiten haben, den Ursprung der Produkte zu deklarieren. Da die Ursprungsregeln in den Handelsabkommen, die zwischen verschiedenen RCEP-Mitgliedsländern zuvor bestanden, unterschiedlich waren, stellt die Konsolidierung und Harmonisierung dieser Regeln eine erhebliche Effizienzsteigerung und Kosteneinsparung für die Unternehmen dar. Zusammen mit den Präferenzzöllen können Unternehmen auf dem regionalen Markt wettbewerbsfähiger werden, einen größeren Kundenkreis erreichen, mehr Gewinn erzielen und somit ihr Geschäft ausweiten. Daher werden von den niedrigeren Handelsbarrieren zwischen den RCEP-Ländern erhebliche handelsschaffende Effekte erwartet.

Die geografische Nähe ist einer der größten Vorteile, die das RCEP-Abkommen für Australien und Neuseeland mit sich bringt, da diese beiden Länder von den meisten Teilen der Welt weit entfernt sind. Wenn das RCEP-Abkommen in Kraft tritt, können australische und neuseeländische Unternehmen neben Präferenzzöllen auch von niedrigeren Transportkosten profitieren, indem sie ihre derzeitigen Lieferanten von außerhalb durch neue innerhalb der Region ersetzen.

Für die sogenannten Plus-Eins-Länder China, Südkorea und Japan ist die Verringerung der Handelsumlenkung durch die ASEAN-Plus-Eins-Freihandelsabkommen ein wichtiger Effekt. Das RCEP-Abkommen ermöglicht daher auch eine Konsolidierung der verschiedenen einzelnen Handelsabkommen zwischen den Mitgliedsländern. Abbildung 1 zeigt, welche Länder bereits vorher Freihandelsabkommen mit anderen RCEP-Mitgliedsländern hatten. Untersucht man, welche Länder vor der Unterzeichnung des RCEP-Abkommens noch keine Handelsabkommen miteinander hatten, erhält man wichtige Er-

2 Die USA haben ihr Interesse bekundet, dem „Comprehensive and Progressive Agreement for Trans-Pacific Partnership“ (CPTPP), früher bekannt als Trans-Pacific Partnership (TPP), wieder beizutreten. Dennoch wurden hierzu bisher keine weiteren Maßnahmen ergriffen.

Abbildung 1
**Handelsabkommen innerhalb des RCEP-Blocks**

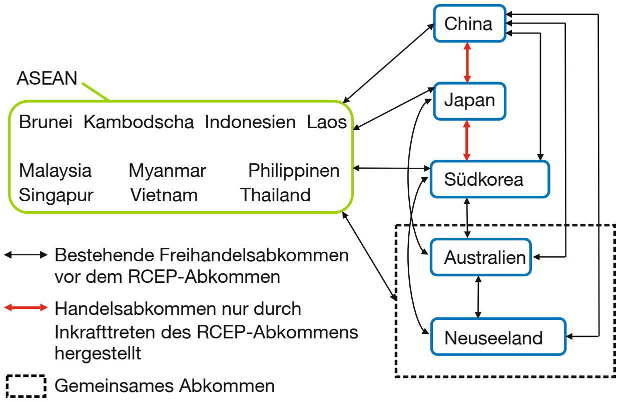

Quelle: eigene Darstellung.

Abbildung 2
**Handelsgruppen im asiatisch-pazifischen Raum**

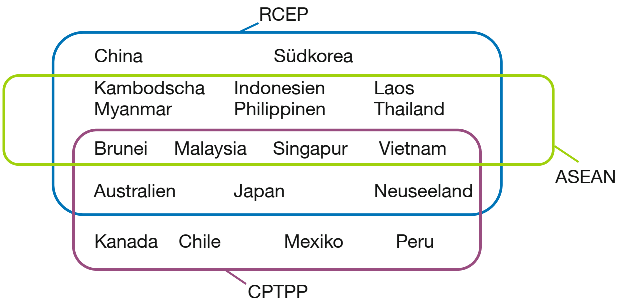

Quelle: eigene Darstellung.

kenntnisse zu der Frage, welche Länder am meisten profitieren. Alle ASEAN-Länder hatten bereits ein Freihandelsabkommen untereinander und die ASEAN-Gruppe hatte Handelsabkommen mit allen anderen RCEP-Mitgliedsländern. Wie Abbildung 1 zeigt, bestehen die neuen Verbindungen nunmehr zwischen China und Japan sowie zwischen Japan und Südkorea. Das bedeutendste neue Handelsabkommen, gemessen an den Handelsströmen, ist die Verbindung, die das RCEP-Abkommen zwischen China und Japan herstellt. Tatsächlich kann das RCEP-Abkommen als ein „China-Japan-Plus"-Handelsabkommen angesehen werden.

Insgesamt wird erwartet, dass die regionale Handelsliberalisierung, die durch das RCEP-Abkommen ermöglicht wird, die Produktivität und Effizienz der Unternehmen in der Region verbessert und das nationale Wirtschaftswachstum ankurbelt (Li et al., 2017; Amidi und Majidi, 2020). Obwohl eine gewisse Handelsumlenkung für einige RCEP-Länder nicht ausgeschlossen werden kann, dürften die Gewinne aus der Handelsschaffung eindeutig überwiegen und zu einem höheren Wirtschaftswachstum in den Mitgliedsländern führen. Petri und Plummer (2020) gehen davon aus, dass der durch das RCEP-Abkommen geschaffene Handel wahrscheinlich einen Teil der Handelsströme ersetzen wird, die durch den Handelskrieg zwischen den USA und China zerstört wurden. Mittels eines berechenbaren allgemeinen Gleichgewichtsmodells schätzen sie, dass sich die Gewinne des RCEP-Abkommens für die Mitgliedsländer bis zum Ende des Jahrzehnts auf 174 Mrd. US-$ belaufen werden, was 0,4 % ihres BIP entspricht. Die größten Gewinne werden für China, Japan und Südkorea erwartet, nicht nur weil diese Länder die größten Länder des Abkommens sind, sondern auch weil der Freihandel der kleineren Länder bereits durch andere Handelsabkommen gesichert ist. Dynamische Effekte, die sich aus der erhöhten Attraktivität der Region für Investitionen ergeben, werden die Wachstumseffekte langfristig zusätzlich verstärken.

## Das RCEP- und das CPTPP-Abkommen

Das Comprehensive and Progressive Agreement for Trans-Pacific Partnership (CPTPP) ist ein Freihandelsabkommen, das im März 2018 zwischen elf Ländern im pazifischen Raum unterzeichnet wurde. Das CPTPP-Abkommen wurde aus der Transpazifischen Partnerschaft (TPP) entwickelt, nachdem sich die USA im Januar 2017 aus dem TPP-Abkommen zurückgezogen hatten. Auf die CPTPP-Mitgliedsländer entfallen zusammen 13,3 % des weltweiten BIP, 6,7 % der Weltbevölkerung und 14,4 % des Welthandels im Jahr 2020 (Australian Department of Foreign Affairs and Trade, 2020b).

Es gibt einige Überschneidungen bei den unterzeichnenden Ländern zwischen dem RCEP- und dem CPTPP-Abkommen, nämlich Australien, Brunei, Japan, Malaysia, Neuseeland, Singapur und Vietnam (vgl. Abbildung 2). Das Fehlen der am wenigsten entwickelten Länder ermöglichte es dem CPTPP-Abkommen, höhere und strengere Standards für nachhaltige Entwicklung und den Schutz von Arbeitsrechten anzustreben und gleichzeitig den Handel zu liberalisieren. Darüber hinaus konnte das CPTPP-Abkommen durch die Abwesenheit der beiden größten Volkswirtschaften der Welt, China und USA, relativ viele Handelshemmnisse zwischen den Mitgliedsländern beseitigen. Dies gilt insbesondere für den Abbau von nichttarifären Maßnahmen, die vielfach ein großes

Abbildung 3
**Bedeutung des internationalen Handels der EU mit den RCEP-Mitgliedsländern**

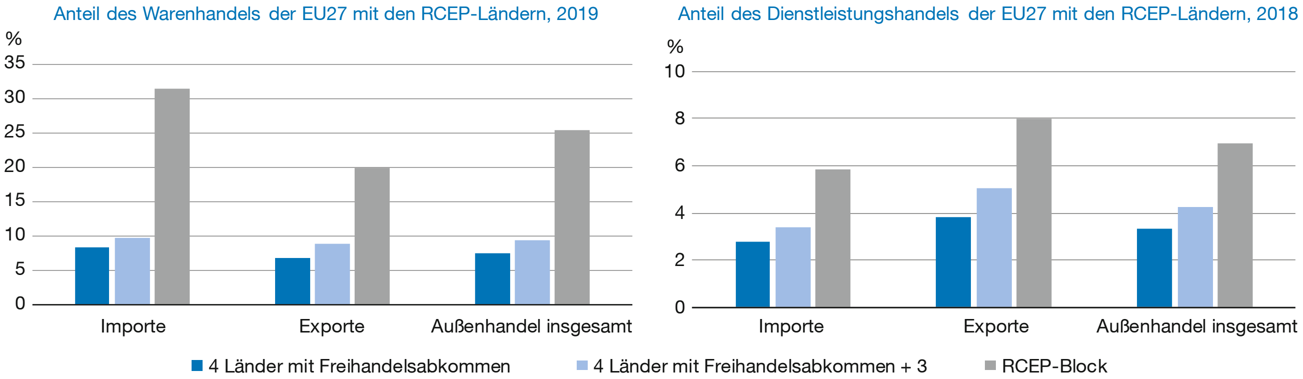

Anmerkungen: „4 Länder mit Freihandelsabkommen" umfassen Japan, Südkorea, Singapur und Vietnam; „4 Länder mit Freihandelsabkommen + 3" umfassen zusätzlich Australien, Indonesien und Neuseeland. Angaben beziehen sich auf die zuletzt verfügbaren Daten.

Quelle: Europäische Kommission; eigene Darstellung.

Hindernis für die regionale Handelsliberalisierung darstellen. Obwohl die USA und China ihr Interesse an einem Beitritt zum CPTPP-Abkommen bekundet haben, wird China nach dem erfolgreichen Abschluss des RCEP-Abkommens bei den Verhandlungen mit dem CPTPP-Block vermutlich einen Vorteil gegenüber den USA haben.

Es ist bemerkenswert, dass das Vereinigte Königreich am 1. Februar 2021 – einen Monat nach dem Ende der Brexit-Übergangszeit – einen formellen Antrag auf CPTPP-Mitgliedschaft gestellt hat. Die ersten Verhandlungen werden voraussichtlich innerhalb weniger Monate beginnen. Wenn die Verhandlungen positiv verlaufen, wird dies die Chance für das Vereinigte Königreich erhöhen, ein Handelsabkommen mit den USA zu erreichen, da Präsident Biden im Januar 2021 angedeutet hat, dass er sich um Neuverhandlungen mit den Teilnehmerländern des CPTPP-Abkommens bemühen würde.

## Die Auswirkungen des RCEP-Abkommens auf die internationalen Handelsströme der EU

Die EU hat bilaterale Freihandelsabkommen mit vier RCEP-Mitgliedsländern: Japan, Südkorea, Singapur und Vietnam. Sie verhandelt derzeit auch über Freihandelsabkommen mit drei weiteren Mitgliedern, nämlich Australien, Neuseeland und Indonesien. Diese bilateralen Freihandelsabkommen reichen jedoch nicht aus, um den Einfluss der EU in der asiatisch-pazifischen Region nach Inkrafttreten des RCEP-Abkommens zu festigen. Abbildung 3 zeigt, dass 2019 alle Mitgliedsländer des RCEP-Abkommens zusammen 25,5 % des gesamten Warenhandels der EU ausmachten. Genauer gesagt entfielen auf die Importe aus den RCEP-Mitgliedsländern 31,5 % der gesamten EU-Importe, und auf die Exporte in die RCEP-Länder entfielen etwa 20 % der EU-Exporte. Die Anteile des Handels mit Dienstleistungen sind deutlich geringer: Auf die RCEP-Länder entfielen etwa 6 % der Dienstleistungsimporte und 8 % der Dienstleistungsexporte. Mit der fortschreitenden Digitalisierung dürften diese Anteile jedoch in den nächsten Jahren an Bedeutung gewinnen.

Bisher war die EU im asiatisch-pazifischen Raum vor allem bei technisch anspruchsvollen Gütern wettbewerbsfähig. Mit reduzierten Handelsbarrieren innerhalb der RCEP-Region könnte die EU Gefahr laufen, unter Handelsumlenkungen zu leiden, wenn ihre Exporte in die RCEP-Länder tatsächlich teurer werden als Waren aus der RCEP-Region und somit durch den Handel innerhalb der RCEP-Region ersetzt werden. Dies lässt sich am Beispiel der Exporte der EU nach China veranschaulichen. China ist der zweitgrößte Exportmarkt der EU. Vor Abschluss des RCEP-Abkommens hatten sowohl Japan als auch die EU keine Freihandelsabkommen oder ein gemeinsames regionales Handelsabkommen mit China. Die durch das RCEP-Abkommen ermöglichten Präferenzzölle könnten die chinesischen Importströme von der EU nach Japan umlenken, da Japan in China um die gleiche Art von qualifikationsintensiven Gütern konkurriert. Bei EU-Exporten in die vier RCEP-Mitgliedsländer, mit denen die EU bereits ein Freihandelsabkommen geschlossen hat, ist dieser Effekt unwahrscheinlich.

Insgesamt werden die Vorzugszölle Anreize für Unternehmen der RCEP-Region schaffen, zu regionalen Lieferanten zu wechseln. Es ist zu erwarten, dass niedrigere Transportkosten innerhalb der RCEP diese Entwicklung unterstützen werden. Wenn die EU keine geeignete Methode zur Bewältigung dieser Herausforderungen findet, wird ihre preisliche Wettbewerbsfähigkeit, die bisher sehr wichtig für ihre gesamten Handelsaktivitäten war, wahrscheinlich in ganzen Bereichen sinken. Ein Beispiel ist die Automobilindustrie. Das RCEP-Abkommen kann erhebliche Auswirkungen auf europäische Produzenten, insbesondere aus Deutschland, haben, da das Handelsabkommen insbesondere Toyota, Honda, Nissan, Hyundai und Kia einen leichteren Zugang zu den Märkten der RCEP-Mitgliedsländer ermöglicht.

Nicht alle Auswirkungen des RCEP-Abkommens auf die EU müssen negativ sein. Es sind zumindest drei positive indirekte Effekte zu erwarten. *Erstens* profitieren auch europäische Unternehmen mit ihren Direktinvestitionen in der Region von der Harmonisierung und Konsolidierung der Ursprungslandregeln. *Zweitens* werden die Unternehmenden in den RCEP-Mitgliedsländern aufgrund sinkender Handelsbarrieren innerhalb der Region mit mehr Wettbewerb aus anderen Mitgliedsländern konfrontiert. Dies übt Druck auf diese Unternehmen aus, ihre Effizienz zu steigern, und schafft Möglichkeiten, Skaleneffekte zu erzielen, wodurch die Importpreise der EU aus dieser Region sinken können. In dem Maße, in dem dies auf Zwischenprodukte von EU-Firmen zutrifft, wird dies deren Produktionskosten senken und ihre preisliche Wettbewerbsfähigkeit erhöhen. *Drittens* kann davon ausgegangen werden, dass die regionale Handelsliberalisierung das Einkommen und das Wachstum der RCEP-Mitgliedsländer erhöht, was zu einer höheren Nachfrage nach höherwertigen Waren und Dienstleistungen führt. Dieser Einkommenseffekt kann europäische Exporteure mit komparativen Vorteilen bei hochwertigen Gütern und Dienstleistungen in der RCEP-Region stärken.

## Konsequenzen für die zukünftige EU-Handelspolitik

Die vorangegangenen Abschnitte legen nahe, dass das RCEP-Abkommen erhebliche Herausforderungen für die Volkswirtschaften der EU mit sich bringen kann. Sie ergeben sich aus der Verringerung der Bedeutung europäischer Standards auf globaler Ebene und aus Handelsumlenkungseffekten. Sie könnten Auswirkungen auf die internationalen Handelsströme der EU und auf ausländische Direktinvestitionen haben. Die EU ist eine der wichtigsten Herkunftsgebiete ausländischer Direktinvestitionen der ASEAN-Länder gewesen. Vor diesem Hintergrund gibt es insbesondere die folgenden vier strategischen Optionen für die Reaktion der EU auf das RCEP-Abkommen:

1. Die EU verstärkt ihre Bemühungen nicht über das hinaus, was bereits eingeleitet wurde. Derzeit hat die EU jeweils ein Freihandelsabkommen mit Japan, Südkorea, Singapur und Vietnam, und sie verhandelt über Handelsabkommen mit Australien, Indonesien und Neuseeland. Diese Abkommen könnten die Handelsumlenkung reduzieren, würden die EU aber immer noch außerhalb des RCEP-Raums lassen.

2. Die EU könnte ein Partnerschaftsabkommen mit dem RCEP-Block anstreben. Wenn es zustande käme, wäre es ein Abkommen zwischen den beiden größten Handelsabkommen der Welt. Es würde den freien Marktzugang der EU zu den Mitgliedsländern des RCEP-Abkommens sichern. Eine kleinere Alternative wäre, eine Partnerschaft mit ASEAN einzugehen, da die EU bereits Handelsabkommen mit mehreren der anderen Mitgliedsländer des RCEP-Abkommens hat. Was dann übrig bliebe, wäre ein Handelsabkommen mit China.

3. Die EU könnte versuchen, sich mit den USA und Indien zusammenzuschließen, um einen alternativen Handelsblock zu bilden. Ein Bündnis mit diesen beiden Ländern, die sich in der jüngeren Vergangenheit als primär nationalistisch ausgerichtet erwiesen haben, birgt jedoch Risiken. Die Handelspolitik der USA unter dem früheren Präsidenten Trump hat dazu geführt, dass diese Option für viele Regierungen der EU-Mitgliedsländer weniger attraktiv wurde.

4. Die EU könnte sich Partner suchen, um die WTO zu reformieren und einen liberaleren Handel auf globaler Ebene zu etablieren. Dies würde bedeuten, die Doha-Runde von 2001 zu reaktivieren oder zu modifizieren, die seit geraumer Zeit praktisch zum Stillstand gekommen ist. Im Erfolgsfall hätte ein solcher Ansatz den Vorteil, dass die handelsschaffenden Effekte auf globaler Ebene maximiert und die handelsumlenkenden Effekte regionaler Abkommen minimiert werden. Die Nachteile dieser Strategie liegen auf der Hand: Sie würde nicht nur zusätzliche Schritte in Richtung eines global liberalisierten Handels erfordern, sondern auch eine Reform der WTO. Beides würde erhebliche Zeit in Anspruch nehmen und selbst dann wäre nicht garantiert, dass ein neues Abkommen zustande kommt. Daher ist diese strategische Option möglicherweise nicht realistisch.

## Fazit

Die Unterzeichnung des RCEP-Abkommens im November 2020 ist ein weiterer Indikator für die zunehmende Bedeutung Asiens in der Weltwirtschaft. Auf die RCEP-Region entfällt ein Viertel des internationalen Handels mit Waren und Dienstleistungen, ein Drittel des weltweiten BIP und fast der gleiche Anteil an der Weltbevölkerung. Sobald das RCEP-Abkommen mit der Ratifizierung einer ausreichenden Zahl von Mitgliedsländern abgeschlossen ist, wird es das größte Freihandelsabkommen der Welt sein. Es kann als ein Abkommen angesehen werden, durch das insbesondere China und Japan, die derzeit zweit- und drittgrößte Volkswirtschaft der Welt, profitieren werden.

Das RCEP-Abkommen kann negative Auswirkungen auf die Wettbewerbsfähigkeit europäischer Unternehmen haben und wird möglicherweise auch die ausländischen Direktinvestitionsströme in die Mitgliedsländer beeinflussen. Außerdem kann es die Bedeutung von EU-Standards bei der Festlegung von Normen im internationalen Handel verringern. Dies wirft die Frage auf, wie die EU auf die Schaffung des RCEP-Raums reagieren sollte. Es gibt mehrere strategische Optionen für die EU, aber keine davon ist ideal oder leicht umzusetzen. Die stetig wachsende Bedeutung der asiatisch-pazifischen Region in der Weltwirtschaft legt jedoch nahe, dass unabhängig davon, für welche Option sich die EU entscheidet, eine engere Allianz mit den RCEP-Ländern geboten erscheint.

**Title:** ***The RCEP and Its Relevance to the EU***

**JEL Classification:** *F02, F15, F53*
